# Triage—clinical reasoning on emergency nursing competency: a multiple linear mediation effect

**DOI:** 10.1186/s12912-024-01919-8

**Published:** 2024-04-24

**Authors:** Won-Oak Oh, Myung-Jin Jung

**Affiliations:** College of Nursing, Korea University, 145 Anam-ro, Seongbuk-gu, 02841 Seoul, South Korea

**Keywords:** Clinical reasoning, Emergency Departments, Emergency nurse, Emergency nursing, Mediation analyses, Triage

## Abstract

**Background:**

Triage is the first step in providing prompt and appropriate emergency nursing and addressing diagnostic issues. Rapid clinical reasoning skills of emergency nurses are essential for prompt decision-making and emergency care. Nurses experience limitations in emergency nursing that begin with triage. This cross-sectional study explored the mediating effect of perceived triage competency and clinical reasoning skills on the association between Korean Triage and Acuity Scale (KTAS) proficiency and emergency nursing competency.

**Methods:**

A web-based survey was conducted with 157 emergency nurses working in 20 hospitals in South Korea between mid-May and mid-July 2022. Data were collected utilizing self-administered questionnaires to measure KTAS proficiency (48 tasks), perceived triage competency (30 items), clinical reasoning skills (26 items), and emergency nursing competency (78 items). Data were analyzed using the PROCESS macro (Model 6).

**Results:**

Perceived triage competency indirectly mediate the relationship between KTAS proficiency and emergency nursing competency. Perceived triage competency and clinical reasoning skills were significant predictors of emergency nursing competency with a multiple linear mediating effect. The model was found have a good fit (F = 8.990, *P* <.001) with, a statistical power of 15.0% (R² = 0.150).

**Conclusions:**

This study indicates that improving emergency nursing competency requires enhancing triage proficiency as well as perceived triage competency, which should be followed by developing clinical reasoning skills, starting with triage of emergency nurses.

**Supplementary Information:**

The online version contains supplementary material available at 10.1186/s12912-024-01919-8.

## Introduction

Patients of all ages with varied degrees of clinical urgencies and severities visit emergency departments, and most of them are undiagnosed and unclassified upon arrival [1]. Growing congestion in the emergency department poses a potential risk to the quality and safety of patient care [2]. Since the outbreak of the COVID-19 pandemic, the total number of patients visiting the emergency department has decreased due to fears of infection [3]; however, paradoxically, it is becoming increasingly crowded with non-urgent patients [4]. These issues have led to insufficient provision of appropriate treatment, increased mortality, and reduced patient satisfaction [5]. The Korean Triage and Acuity Scale (KTAS) is consistent with the 5 stages (Level 1: resuscitation ∼ Level 5: non-urgent) defined by the Canadian Triage and Acuity Scale (CTAS) and is divided into adult and pediatric areas based on the criterion of age 15 [6].

Triage is the initial step in emergency nursing, in which patients are classified based on their urgency and severity, determining the priority of treatment, and enabling efficient emergency interventions [7]. Globally, 76.5–91% of triages are performed by general nurses in emergency departments [8, 9]. However, triage errors, such as over- or under-triaging, can occur and potentially increase disease severity and mortality rates [7, 10]. Therefore, case-based education is regularly conducted using the KTAS program to enhance triage accuracy [11, 12]. As a result, triage proficiency is considered essential for emergency nurses. Daily auditing and monitoring have been shown to reduce triage error rates and improve consistency with doctors’ opinions as well as proficiency in triage [13]. This underscores the importance of continuous experiential education for nurses pursuing professional growth.

However, studies have shown that emergency nurses often perceive their triage proficiency as low, and that their accuracy does not significantly improve even after learning triage scales [14, 15]. Moreover, nurses were not accurately aware of what triage competencies they should possess [9, 16]. Triage competency in emergency nursing extends beyond mere triage accuracy to include patient disposition, appropriate emergency treatment, impression assessment, and re-evaluation, representing a broad concept [9, 17]. In this process, emergency nurses assess the urgency of care and become primary decision-makers in patient care planning [16].

Accurate decision-making based on rapid clinical reasoning should take precedence in emergency care starting with triage in emergency departments [18]. Clinical reasoning skills are a major factor affecting emergency nursing competency (ENC) [18, 19]. Previous studies have shown that clinical reasoning skills improve after learning triage scales [20]. Furthermore, it has been revealed that triage competency can be a contributing factor to ENC [21]. Professional self-concept is an important factor in determining triage competency among emergency nurses [1]. However, the current education program focuses only on triage accuracy, that is, KTAS proficiency [7, 10, 22]. KTAS proficiency refers to the ability to quickly determine the KTAS level by selecting the most appropriate chief complaint category for the patient’s symptoms presented using the KTAS program.

Considering the importance of accurate decision-making based on swift clinical reasoning after triage in emergency nursing practice, it is necessary to identify the relationship between factors that influence ENC [20, 23]. Therefore, a potential causal relationship among KTAS proficiency, perceived triage competency, clinical reasoning skills, and ENC can be inferred; however, there is a lack of research clearly identifying these relationships [16–23].

**Fig. 1:**
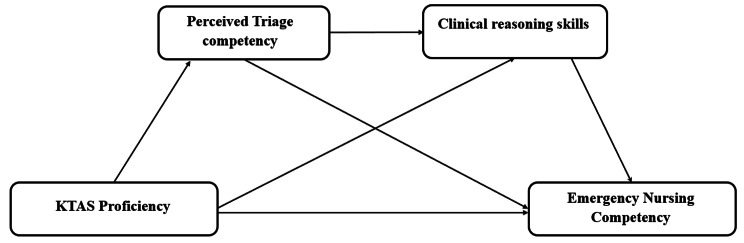
Research hypothesis framework of multi-mediation model

This study began with the assumption that emergency nurses with a limited perception of triage, lack clinical reasoning skills [23]. This study aimed to analyze the multiple mediating effects of perceived triage competency and clinical reasoning skills on the relationship between KTAS proficiency and ENC. The research model and hypotheses are as follows (Fig. 1):

### Research hypotheses

First, KTAS proficiency has an effect on ENC.

Second, KTAS proficiency impacts ENC through the mediation of perceived triage competency.

Third, KTAS proficiency impacts ENC through the mediation of clinical reasoning skills.

Fourth, perceived triage competency and clinical reasoning skills mediate the impact of KTAS proficiency on ENC.

## Methods

This study employed a cross-sectional design. The online survey (Google Docs) was conducted from May 22, 2022, to July 21, 2022, in South Korea, until the minimum number of certified emergency unit nurses could join. A total of 157 emergency nurses participated in self-administered questionnaires, including 37 nurses at six regional emergency medical centers and 120 nurses at 14 local emergency medical centers. The relationships between KTAS proficiency, perceived triage competency, clinical reasoning skills, and ENC were analyzed using the PROCESS macro (Model 6) in SPSS version 3.4 [24].

### Participants

The study participants were emergency nurses working in emergency departments in South Korea, where the KTAS was implemented. However, nurses with less than 12 months of clinical experience in the emergency department [25] and those who only performed administrative tasks without direct patient care were excluded from the selection criteria [11].

The sample size for this study was calculated using the G-power 3.1.9.2 program, with effect size (d = 0.15), significance level (α = 0.05), power (*β* = 0.95), and three predictor variables entered as input. The effect size of the study was calculated using the median effect size in a multiple regression analysis (${f^2} = \frac{{{R^2}}}{{1 - {R^2}}}$) (Cohen, 1988) [26] based on the results of previous studies [27]. A total of 157 emergency nurses were recruited, taking into account a dropout rate of approximately 30%, and no subjects were excluded from the final analysis.

### Measurements

Self-administered questionnaires were used to assess emergency nurses’ KTAS proficiency, perceived triage competency, clinical reasoning skills, and ENC. The general characteristics of the emergency nurses were measured using a questionnaire. The questionnaire included items concerning demographic (nine items) and KTAS-related characteristics (two items).

#### KTAS proficiency

KTAS is composed of 17 major and 166 subcategories based on the main symptoms [6, 11]. In this study, a draft KTAS proficiency questionnaire was developed based on the detailed KTAS [6] & *KTAS - Provider training manual* [11]. KTAS was reorganized into 48 tasks in 7 domains by integrating KTAS program items with the emergency patient classification process and grouping similar items together. The tool was validated using a content validity index (CVI) by a panel of eight experts, consisting of one emergency physician, one nursing professor, two emergency nurse specialists, and four emergency nurses with more than 10 years of emergency department experience [28]. All of whom had a CVI > 0.8 and a content validity ratio (CVR) > 0.88. As a result, KTAS proficiency was categorized into 7 domains and 48 tasks: critical first look (2 tasks), infection control (2 tasks), 1st order modifiers (4 tasks), 2nd order modifiers (8 tasks), special circumstances (3 tasks), adult area (17 tasks), and pediatric area (12 tasks) (see Additional file 1).

Participants rated their KTAS proficiency on a 4-point Likert scale (1 = requiring overall assistance, 2 = requiring some assistance, 3 = capable of independent performance, and 4 = capable of providing education and consultation), with higher scores indicating higher KTAS proficiency. Cronbach’s alpha was 0.96 in the present study.

#### Perceived triage competency

Perceived triage competency was measured using the 30-item Triage Competency Scale for Emergency Nurses [9]. The tool includes five factors: clinical judgment (thirteen items), expert assessment (four items), management of medical resources (four items), timely decision (four items), and communication (five items). Participants rated their perceived triage competency on a 5-point Likert scale (1 = not at all, 5 = always), with higher scores indicating higher perceived triage competency. Cronbach’s alpha was 0.95 in the present study.

#### Clinical reasoning skills

Clinical reasoning skills were measured using a 26-item scale with 6 factors from the Korean version of the Clinical Reasoning Skill Scale [29, 30]. The tool includes six factors: collecting information (five items), processing information (five items), identifying problems/issues (four items), establishing goals (four items), taking action (five items), and evaluating outcomes (three items). Participants rated their clinical reasoning skills on a 5-point Likert scale (1 = very poorly, 5 = excellent), with higher scores indicating higher clinical reasoning skills. Cronbach’s alpha was 0.96 in the present study.

#### Emergency nursing competency

ENC was measured using the 78-item Competence Scale of Actions of Nurses in Emergencies (CSANE) [31]. A translation agency and the authors translated the English tool into Korean using translation reverse translation method, and eight experts then confirmed the translation’s appropriateness. The tool includes seven factors: professional practice (33 items), relationships at work (19 items), positive challenge (10 items), targeted action (7 items), constructive attitude (2 items), professional excellence (4 items), and adaptation to change (3 items). Participants rated their ENC on a 5-point Likert scale (1 = not at all competent, 5 = very competent), with higher scores indicating higher ENC. Cronbach’s alpha was 0.98 in the present study.

### Statistical data analysis

The data collected were analyzed using IBM SPSS Statistics for Windows (version 25.0; IBM Corp., Armonk, NY). The relationships among the variables were analyzed using Pearson’s correlation coefficients. The multiple mediating effects of perceived triage competency and clinical reasoning skills on the relationship between KTAS proficiency and ENC were analyzed using the PROCESS macro (Model 6) for SPSS version 3.4 [24]. Bootstrapping analysis with 5000 resamples was conducted to test the significance of the mediation effects. The significance level was set at *P* <.05. The direct, indirect, and total effects were deemed statistically significant when the results of the 95% confidence interval excluded zero [24].

## Results

### General characteristics of participants

The general characteristics of the study participants are presented in Table 1. The total clinical experience of the 157 emergency nurses was an average of 8.15 ± 5.87 years, and their emergency department experience was 4.06 ± 3.20 years. Most participants were female (*n* = 140, 89.2%), general nurses (*n* = 105, 66.9%), and working in general hospitals (*n* = 117, 74.5%), and emergency type (local emergency medical center; *n* = 120, 76.4%). The KTAS-related characteristics of participants included experience with KTAS education (*n* = 131, 83.4%), and maintenance of KTAS certification (*n* = 101, 64.3%). Education (F = 7.121, *P* =.001) and position (F = 3.860, *P* =.023) had significant effects on ENC (Table 1).

**Table 1:** Sample characteristics and differences in emergency nursing competency (*N* = 157)

Characteristics	Categories	Mean ± SD or n (%)	Emergency nursing competency				
			Mean ± SD	t or F	Sheffe^*^		*P*
Sex	Male	17 (10.8)	3.88 ± 0.51	0.251			0.802
	Female	140 (89.2)	3.85 ± 0.48				
Age (year)		33.31 ± 6.97					
	20 ∼ 29	52 (33.1)	3.81 ± 0.52	0.506			0.604
	30 ∼ 39	75 (47.8)	3.87 ± 0.48				
	≥ 40	30 (19.1)	3.91 ± 0.42				
Marriage status	Single	89 (56.7)	3.83 ± 0.52	− 0.593			0.554
	Married	68 (43.3)	3.88 ± 0.44				
Education	Diploma^a^	27 (17.2)	3.71 ± 0.41	7.121	a, b < c		0.001**
	Bachelor^b^	106 (67.5)	3.82 ± 0.48				
	More than Graduate^c^	24 (15.3)	4.17 ± 0.43				
Clinical experience (year)		8.15 ± 5.87					
	1 ∼ 5	64 (40.8)	3.81 ± 0.46	2.926			0.057
	6 ∼ 10	52 (33.1)	3.79 ± 0.52				
	≥ 11	41 (26.1)	4.01 ± 0.45				
Clinical experience in the ED (year)		4.06 ± 3.20					
	1 ∼ 2	59 (37.6)	3.85 ± 0.47	0.425			0.654
	3 ∼ 5	64 (40.8)	3.82 ± 0.49				
	≥ 6	34 (21.7)	3.92 ± 0.50				
Position	General nurse^a^	105 (66.9)	3.78 ± 0.50	3.860	a < b		0.023*
	Charge nurse^b^	39 (24.8)	4.03 ± 0.43				
	Head nurse^c^	13 (8.3)	3.90 ± 0.32				
Hospital type	General Hospital	117 (74.5)	3.86 ± 0.50	0.460			0.646
	Advanced General Hospital	40 (25.5)	3.82 ± 0.43				
Emergency type	Local Emergency Medical Center	120 (76.4)	3.84 ± 0.48	− 0.279			0.782
	Regional Emergency Medical Center	37 (23.6)	3.87 ± 0.51				
Experience with KTAS education	Have	131 (83.4)	3.86 ± 0.49	0.315			0.753
	None	26 (16.6)	3.83 ± 0.44				
Maintenance of KTAS certification	Yes	101 (64.3)	3.87 ± 0.50	0.589		0.557	
	No or None	56 (35.7)	3.82 ± 0.46				

ED = Emergency department; KTAS = Korean Triage and Acuity Scale; SD = Standard deviation

*P <.05, **P <.01, ***P <.001

### KTAS proficiency

The mean score of KTAS proficiency was 3.05 ± 0.78 out of 4 points. The score was the highest for vital signs (3.39 ± 0.73) in the 1st order modifiers while that for orthopedics (pediatric gait disorder / painful walk) was the lowest (2.80 ± 0.82) in the pediatric area (see Additional file 1).

### Correlations between the variables

The correlations among KTAS proficiency, perceived triage competency, clinical reasoning skills, and ENC are shown in Table 2. ENC was positively correlated with KTAS proficiency (*r* =.314, *P* <.001), perceived triage competency (*r* =.758, *P* <.001), and clinical reasoning skills (*r* =.667, *P* <.001) (Table 2).

**Table 2:** Descriptive statistics of variables and correlations between variables (*N* = 157)

	M ± SD	r (p)				
		Emergency nursing competency	KTAS Proficiency	Perceived Triage competency		Clinical reasoning skills
Emergency nursing competency	3.85 ± 0.48	1				
KTAS Proficiency	3.05 ± 0.46	0.314 < 0.001	1			
Perceived Triage competency	3.88 ± 0.50	0.758 < 0.001	0.314 < 0.001	1		
Clinical reasoning skills	3.88 ± 0.57	0.667 < 0.001	0.265 < 0.001	0.745 < 0.001	1	

### Path model of the multi-mediating effects between the variables

The relationship between KTAS proficiency and ENC was examined using the PROCESS macro (Model 6), with perceived triage competency and clinical reasoning skills as multiple mediators. Education and position were significant background variables affecting ENC, and were adjusted as covariates in the path analysis.

**Fig. 2:**
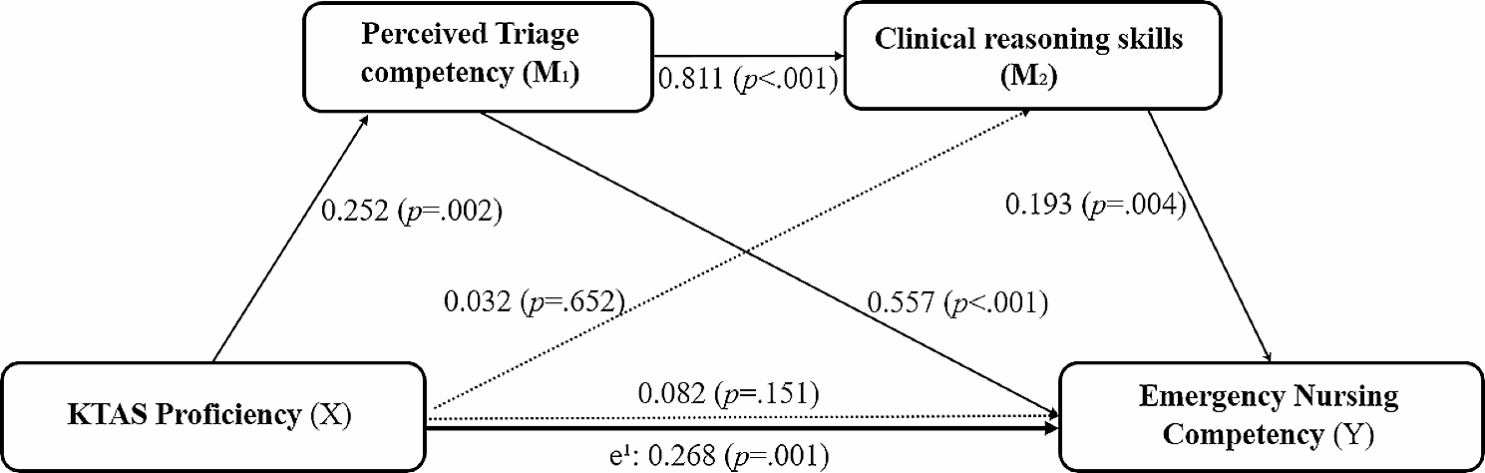
Path model of the multi-mediating effects between the variables Path analysis of KTAS proficiency, Perceived Triage competency, Clinical reasoning skills, and Emergency nursing competency among emergency nurses (*N* = 157). Solid lines represent significant paths, while dashed lines represent non-significant paths. Parameters displayed are standardized coefficients of the direct effect on each pathway, except for the total effect of X on Y (e¹) Note: X: independent variable, Y: dependent variable, M_1_: mediator 1, M_2_: mediator 2, e¹: Total effect of X on Y

First, KTAS proficiency had a significant effect on the mediating variable of perceived triage competency (β = 0.252, *P* =.002) (Fig. 2). The model was found to have a good fit (F = 16.910, *P* <.001), with a statistical power of 9.8% (R² = 0.098). The control variable education had a significant effect on perceived triage competency (β = 0.238, *P* <.001).

Second, KTAS proficiency did not have a direct significant effect on clinical reasoning skills (β = 0.032, *P* =.652). However, it was found that perceived triage competency as mediator variable 1 had a significant effect on the clinical reasoning skills as mediator variable 2 (β = 0.811, *P* <.001) (Fig. 2). The model was found to have a good fit (F = 48.551, *P* <.001), with a statistical power of 56.1% (R² = 0.561). However, education and position, which were the control variables, did not significantly affect clinical reasoning skills.

Third, KTAS proficiency as an independent variable did not have a direct significant effect on ENC as a dependent variable (β = 0.082, *P* =.151), while perceived triage competency had a significant effect on ENC (β = 0.557, *P* <.001), and clinical reasoning skills had a significant effect on ENC (β = 0.193, *P* =.004) (Fig. 2). These results can be seen in Fig. 2, where the total effect of KTAS proficiency on ENC was statistically significant (β = 0.268, *P* =.001). The model was found to have a good fit (F = 8.990, *P* <.001) with, a statistical power of 15.0% (R² = 0.150). However, education and position, which were the control variables, did not significantly affect ENC.

### Multiple mediating effects with variables

To investigate the multiple mediating effects of perceived triage competency and clinical reasoning skills on KTAS proficiency and ENC, the variables were examined by conducting bootstrapping with 5,000 iterations and setting the confidence interval at 95%. The 95% confidence intervals for the paths “KTAS proficiency -> perceived triage competency -> clinical reasoning skills -> ENC” was 0.004 to 0.115, respectively, indicating significant mediating effects as they were all above zero (Table 3). The causal relationship between KTAS proficiency and ENC clearly indicated the multiple linear mediating effect of perceived triage competency and clinical reasoning skills (Fig. 2).

**Table 3:** Multiple mediating effects results with variables

Division	Pathway	B	S.E.	95% CI
Direct effect of X on Y	KTAS Proficiency → ENC	0.082	0.057	− 0.0304 ∼ 0.1950
Indirect effect(s) of X on Y:	KTAS Proficiency → Perceived Triage competency → ENC	0.140	0.060	0.0336 ∼ 0.2615
	KTAS Proficiency → Clinical reasoning skills → ENC	0.006	0.013	− 0.0154 ∼ 0.0389
	KTAS Proficiency → Perceived Triage competency → Clinical reasoning skills → ENC	0.039	0.029	0.0044 ∼ 0.1150
Total effect of X on Y	KTAS Proficiency → ENC	0.268	0.080	0.1091 ∼ 0.4265

CI: Confidence Interval, ENC: Emergency Nursing Competency, KTAS: Korea Triage Acuity Scale, *S.E.*: Standard error, X: KTAS Proficiency, Y: ENC

## Discussion

The results revealed a multiple linear mediating effect of perceived triage competency and clinical reasoning skills on KTAS proficiency and ENC. However, KTAS proficiency did not have a direct impact on ENC, and perceived triage competency acted as a complete mediator. These findings highlight the significant implications of perceived triage competency and clinical reasoning skills in emergency nursing, starting with triage [7, 10]. Moreover, the education level and position of emergency nurses were significant factors influencing ENC. This finding implies that continuous professional education and training are essential to enhance ENC.

Recent developments in the field of emergency medicine have demonstrated the potential of artificial intelligence (AI) systems in improving triage accuracy [32–35]. However, even Chat-GPT using a large language model (LLM) has limitations of low reliability and stability. It was not effective in replacing human experts such as triage nurses [35]. Agreement with human experts was low, with a higher tendency for over-triage [35]. Predictive screening by machine learning, based on extensive clinical data, could aid healthcare professionals in making decisions [33, 34]; However, unresolved issues of triage errors, including over- and under-triage persist in the emergency department [32, 33]. These issue may stem from the inherent complexity and uncertainty of emergency department triage, which is markedly different from those in emergency department medical evaluations that rely on diagnostic investigations [35]. A discussion based on the main results of the research hypotheses is as follows.

First, no hypothesis was established that KTAS proficiency affects ENC. The initial assessment and prompt treatment response during the first encounter between patients and healthcare providers in the emergency department are of utmost importance [36]. Despite previous research findings indicating that perceived triage competency, clinical reasoning skills, and ENC have significant impacts on patient outcomes, current triage education tends to focus solely on triage accuracy and program proficiency. Recently, research on simulation-based education programs for triage targeting emergency nurses has been conducted in the adult [20, 22, 37–40], and pediatric areas [21, 41]. However, the majority of these education programs focus primarily on triage accuracy and proficiency and result in limited overall improvement in ENC [22]. Thus, the current triage educational program may not be enough to increase ENC.

Second, perceived triage competency was a complete mediating factor between KTAS proficiency and ENC. Triage competency is more than just triage accuracy which is prioritized based on patient urgency. It is not a separate process, but a comprehensive concept of clinical judgment that encompasses professional assessment, medical resource management, timely decision-making, and communication [9, 13]. In other words, failure to properly re-triage, such as re-disposition, appropriate emergency care, impression assessments, and performing reevaluations, could be considered a lack of triage competency, especially in the context of rapidly changing patient conditions. The findings of this study support the need to improve professional self-perception of triage competency to enhance ENC.

Third, clinical reasoning skills had no mediating effects on KTAS proficiency and ENC. Clinical reasoning skills refer to the critical thinking and judgment process through which nurses diagnose potential patient problems and make clinical decisions for problem-solving [42]. Emergency nurses’ perception of trigae was associated with clinical reasoning skills [23, 43]. However, in this study, the mediating effect of clinical reasoning skills on the effect of KTAS proficiency on ENC was not verified.

Fourth, perceived triage competency and clinical reasoning skills were linearly mediated by the effect of KTAS proficiency on ENC. Triage in the emergency department is the first step in determining the urgency and severity of the patient’s condition [5, 16], after which nurses utilize clinical reasoning skills to make clinical judgments and provide emergency nursing care [44]. After evaluating the critical first look, chief complaint and 1st or 2nd order modifiers, triage nurses determine the triage stage based on the initial impression of the patient [18]. During this process, they apply empirical knowledge and clinical reasoning skills to collaborate on patient disposition and emergency treatment. However, emergency nurses are often trained in clinical reasoning skills separately from triage, and the trainings are mostly universal for novice nurses in emergency nursing duties. The findings of this study support the need to develop clinical reasoning skills starting from triage to enhance ENC.

The results showed that perceived triage competency and clinical reasoning skills, starting with triage, had a multiple linear mediating effect on ENC. Therefore, to improve ENC, perceived triage competency and clinical reasoning skills should be developed along with a program for improving the triage proficiency of emergency nurses. In the future, we suggest the development of an educational program aimed at enhancing ENC, starting with triage.

### Limitations

Despite the significant results, this study has several limitations. A cross-sectional survey was conducted in a limited online environment in a single country. To generalize these findings, we propose the development of triage clinical reasoning programs that can be globally validated.

## Conclusion

To improve ENC in the field, efforts are needed to enhance perceived triage competency and clinical reasoning skills in emergency nursing, starting with triage. The results showed that KTAS proficiency was not a direct influencing factor of ENC and that perceived triage competency was an important mediating predictor. Perceived triage competency and clinical reasoning skills had a multiple linear mediating effect on KTAS proficiency and ENC. Comprehensively, we expressed the need for clinical reasoning skills, starting with triage, to improve emergency nursing competencies.

## Implications

This study provides new evidence for KTAS proficiency training for current triage accuracy and key insights into what affects ENC. Although KTAS proficiency did not directly affect ENC, perceived triage competency was completely mediated. However, KTAS proficiency did not affect clinical reasoning skills. Finally, perceived triage competency and clinical reasoning skills had a multiple linear mediating effect on ENC. This suggests that clinical reasoning education starting with triage is needed rather than the current education that focuses on KTAS proficiency. This can guide targeted interventions and educational programs to enhance the skills and competencies of nurses in emergency departments.

### Electronic supplementary material

Below is the link to the electronic supplementary material.

**Supplementary Material 1: Table 1:** KTAS proficiency questionnaire consisting of 7 domains and 48 tasks

## Data Availability

All the data generated or analyzed during this study have been included in the published article and its accompanying supplementary information files.
