# Supplementary material for: Triage—clinical reasoning on emergency nursing competency: a multiple linear mediation effect
Source: BMC Nurs. 2024 Apr 24;23:274. doi: 10.1186/s12912-024-01919-8 (PMC11044571; doi:10.1186/s12912-024-01919-8)
Supplement: Supplementary file 1 — Supplementary Material 1: Table 1: KTAS proficiency questionnaire consisting of 7 domains and 48 tasks [file 12912_2024_1919_MOESM1_ESM.pdf]

Supplementary materials

(Supplementary material table 1). KTAS proficiency (N=157)

Domains (7)	Tasks (48)		Proficiency (Mean ± SD)
Critical first look (2 items)	1	Adult: ABCD assessment (VS: Respiratory Modifiers, Hemodynamic Modifiers, LOC, Temperature)	3.37±0.67
	2	Pediatric Assessment Triangle (overall status, respiratory, circulation)	3.15±0.71
Infection control (2 items)	3	Oral questions or patients themselves	3.27±0.78
	4	Screening for communicable disease	3.29±0.73
1st order modifiers (4 items)	5	Vital Signs	3.39±0.73
	6	Pain Severity Modifier	3.22±0.65
	7	Bleeding disorder	3.34±0.66
	8	Mechanism of Injury	3.16±0.73
2nd order modifiers (8 items)	9	Blood sugar level	3.25±0.80
	10	Emergency level of dehydration	3.01±0.79
	11	Hypertension in adults	3.03±0.78
	12	Chest pain (non-cardiac features)	3.03±0.78
	13	Extremity weakness / Symptoms of CVA	3.22±0.73
	14	Dysphagia/Disorder	3.08±0.76
	15	Injury of upper or lower extremities	3.06±0.75
	16	Waiting patients Re-evaluation	2.98±0.83
Special circumstances (3 items)	17	Environmental Exposure (hypothermia, drowning)	3.05±0.74
	18	Pregnancy issues > 20 wks	2.98±0.91
	19	Mental health and psychosocial issues (depression/suicide/self-harm, anxiety/crisis, hallucinations/delusions)	3.01±0.81
Adult area (17 items)	20	Substance misuse / Intoxication	3.01±0.72
	21	Mental health (Insomnia, violence/murder, social problems, bizarre behavior, abuse, neglect)	2.94±0.75
	22	Neurologic Altered level of consciousness	3.10±0.78
	23	Ophthalmology	3.02±0.76
	24	Nose (nasal congestion / hay fever)	3.01±0.76
	25	Ears (Earache Foreign body ear Loss of hearing Tinnitus)	2.91±0.78
	26	ENT – (Mouth, Throat, Neck, Dental·Gum problems, Facial trauma)	2.92±0.77
	27	Respiratory (Shortness of breath, Respiratory foreign body)	3.07±0.76
	28	Cardiovascular cardiac arrest (non-traumatic)	3.21±0.73
	29	Gastrointestinal (Abdominal pain, Vomiting, Anorexia, Melena)	3.04±0.75
	30	Obstetrics-Gynecology	2.84±0.83
	31	Genito-urinary (Flank pain, Urinary retention, Hematuria, UTI complaints)	2.82±0.85
	32	Orthopedic	2.96±0.75
	33	Trauma (Major trauma – penetrating, blunt)	3.04±0.75
	34	Environmental injury (Frostbite / Cold injury)	2.96±0.74
	35	Skin (Rash, Sting, Redness, Breast abrasion, Stitch-out)	3.05±0.77
	36	General & Minor (Fever, Ring removal, Hyper/hypoglycemia, Abnormal lab values)	3.04±0.75
Pediatric area (12 items)	37	Gastrointestinal (Oral/esophageal foreign body, Neonatal dysphoria, Neonatal jaundice)	2.93±0.76
	38	Mental health (Concern for patient's welfare, Pediatric	2.83±0.84

		disruptive behavior)	
	39	Orthopedics (Pediatric gait disorder / Painful walk)	2.80±0.82
	40	Respiratory modifiers (Stenosis, wheezing, infant apnea)	3.05±0.85
	41	Neurologic (Floppy baby, Hypotonia)	3.05±0.80
	42	General problems (Congenital problem in children, newborn)	2.86±0.93
	43	Interview method considering the age of children	2.84±0.90
	44	Child's symptoms CIAMPEDS classification assessment	2.94±0.84
	45	Vital Signs-Physiological Evaluation	2.98±0.82
	46	Unphysiological indicator (Pain, Hemorrhagic disease, Mechanism of injury)	2.92±0.82
	47	Child's 2nd order modifiers (Blood sugar level, Dehydration)	3.04±0.78
	48	Child's particular C.C (Inconsolable crying in infants, Respiratory foreign body or Oral / Esophageal Foreign Body)	2.97±0.74
Total (Range)			3.05±0.78 (2.80~3.39)
Cronbach' alpha			.96

ABCD: Airway Breath Circulation Disability**, C.C:** Chief Complaint**, CIAMPEDS:** (**C**hief Complaint, Vaccination/**I**solation, **A**llergy, **M**edication, Disease/**P**ast history, **E**vent, **D**iet/Diaper, **S**ymptom record), **CVA:** Cardiovascular accident**, ENT:** Eyes Noses Throat**, LOC:** Level of Consciousness**, UTI:** Urinary tract infection**, VS:** Vital Signs
